# Supplementary material for: Near-lifespan longitudinal tracking of brain microvascular morphology, topology, and flow in male mice
Source: Nat Commun. 2023 May 24;14:2982. doi: 10.1038/s41467-023-38609-z (PMC10205707; doi:10.1038/s41467-023-38609-z)
Supplement: Supplementary file 1 — Supplementary information [file 41467_2023_38609_MOESM1_ESM.pdf]

Supplementary information

Near-lifespan longitudinal tracking of brain microvascular morphology, topology, and flow in male mice

Konrad W. Walek, Sabina Stefan, Jang-Hoon Lee, Pooja Puttigampala, Anna H. Kim, Seong Wook Park, Paul J. Marchand, Frederic Lesage, Tao Liu, Yu-Wen Alvin Huang, David A. Boas, Christopher Moore, Jonghwan Lee

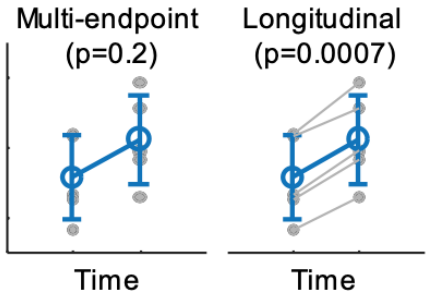

Supplementary Figure 1. An example of the multi-endpoint vs. longitudinal approach. These simulated data demonstrate how identical mean and standard deviations can result in different p-values. The illustrative data were simulated using a simple random number generation in MATLAB. Specifically, we set the means to be 1.0 and 1.5 for the groups 1 and 2, respectively, and then generated random numbers around the mean for each group (n=5), where the random numbers have a standard deviation of 0.5 for both groups and follow the normal distribution. We obtained the p-values via two-sample t-test (left) and paired t-test (right), both two-sided. In using the paired t-test, we assumed that the two groups represent two different time points in a longitudinal measurement.

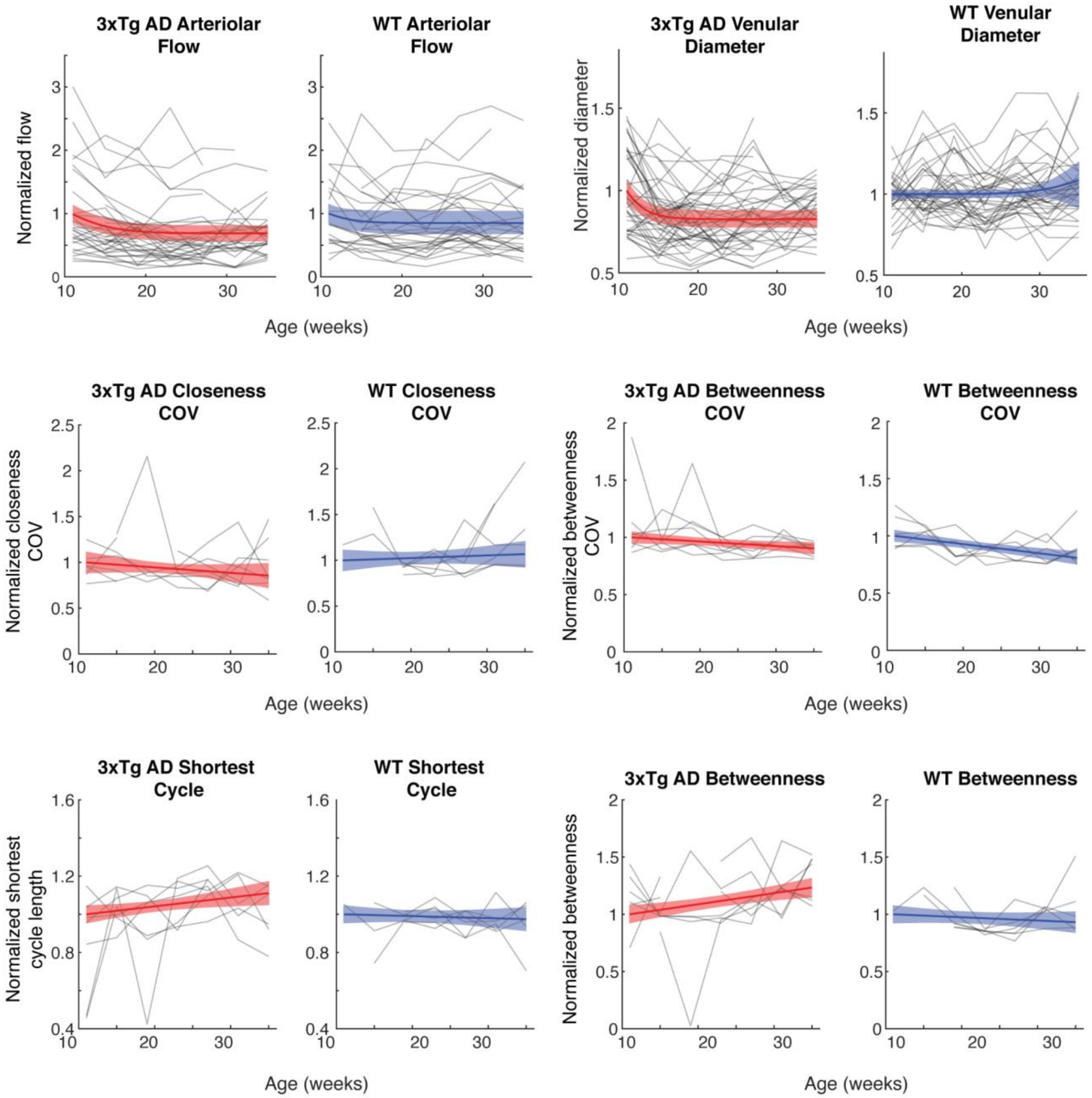

Supplementary Figure 2. Time courses of vascular parameters which were significantly different between 3xTg AD and WT mice. The gray lines indicate individual vessels (arteriolar/venular flow/diameter) or individual animals (other properties). The color lines indicate LME fits and the color shades present the 95% CI.

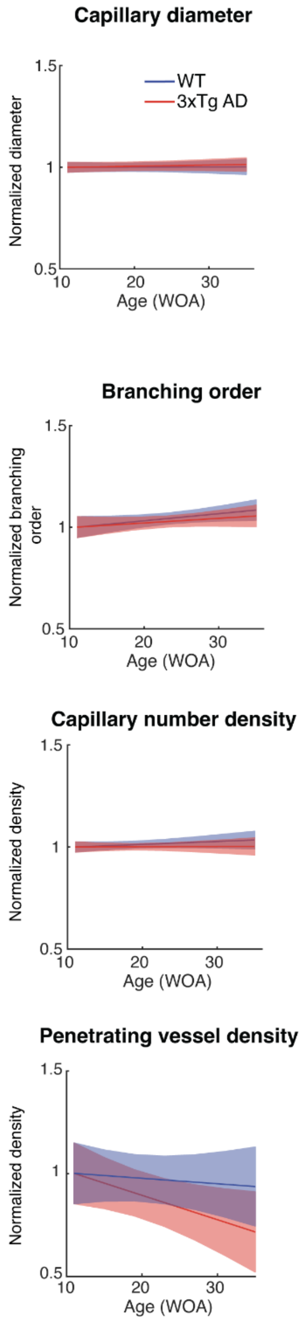

Supplementary Figure 3. All properties which either had statistically no difference in the rates of change between AD and WT or had statistically different rates but the fractional changes did not become statistically different between AD and WT until the end of experiment (35 WOA),

except for the pial vessel diameter which was shown in Fig. 1. The color lines indicate LME fits and the color shades present the 95% CI.

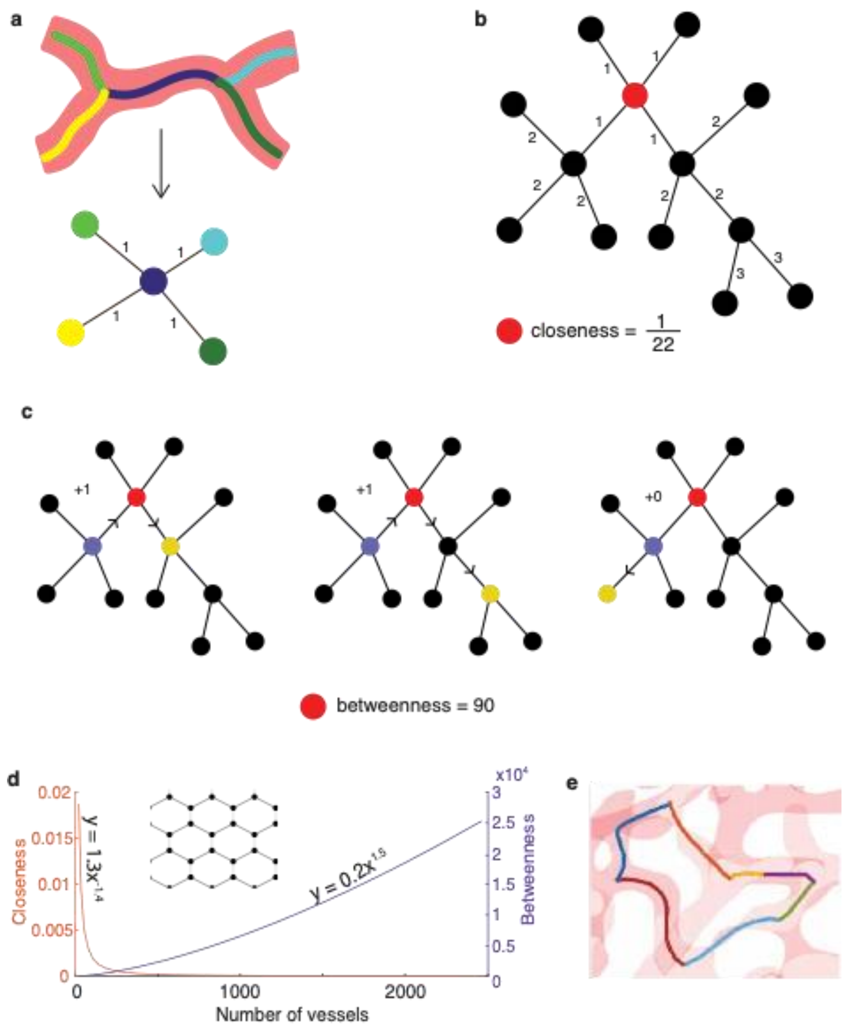

Supplementary Figure 4. Topological properties of a capillary vessel network. Closeness is calculated for each vessel as the reciprocal sum of the number of vessels along the shortest paths between the vessel and every other vessel in the network. Betweenness counts how many times a single vessel is traversed along the shortest path between any other two vessels within the network. The closeness quantifies relative importance of a vessel to the network, and the betweenness indicates the extent to which the network would be vulnerable to malfunction of the vessel. The shortest cycle represents the mean number of vessels traversed in the shortest loop to

return to the same vessel, serving as an indicator of overall connectedness of the vessel. **a)** For conceptual illustration, five vessels are shown, each in a different color, and their representation as nodes and edges. The path length between any two vessels which are directly connected is defined as 1. **b)** An example of how closeness is determined for the red node: the path length for all nodes from the red node are summed and inverted. **c)** Betweenness is an indication of the number of times a node is along the shortest path between any two other nodes: depicted is the scenario when the red node is along the shortest path between the purple and yellow nodes (left two diagrams) and the scenario when the red node is not along the shortest path (right diagram). **d)** The relationship of mean betweenness and closeness with respect to the number of vessels in a capillary network idealized as a honeycomb network. **e)** The illustration shows an example of the shortest cycle for one capillary. This is defined as the smallest number of capillaries that need to be traversed before returning to the starting point.

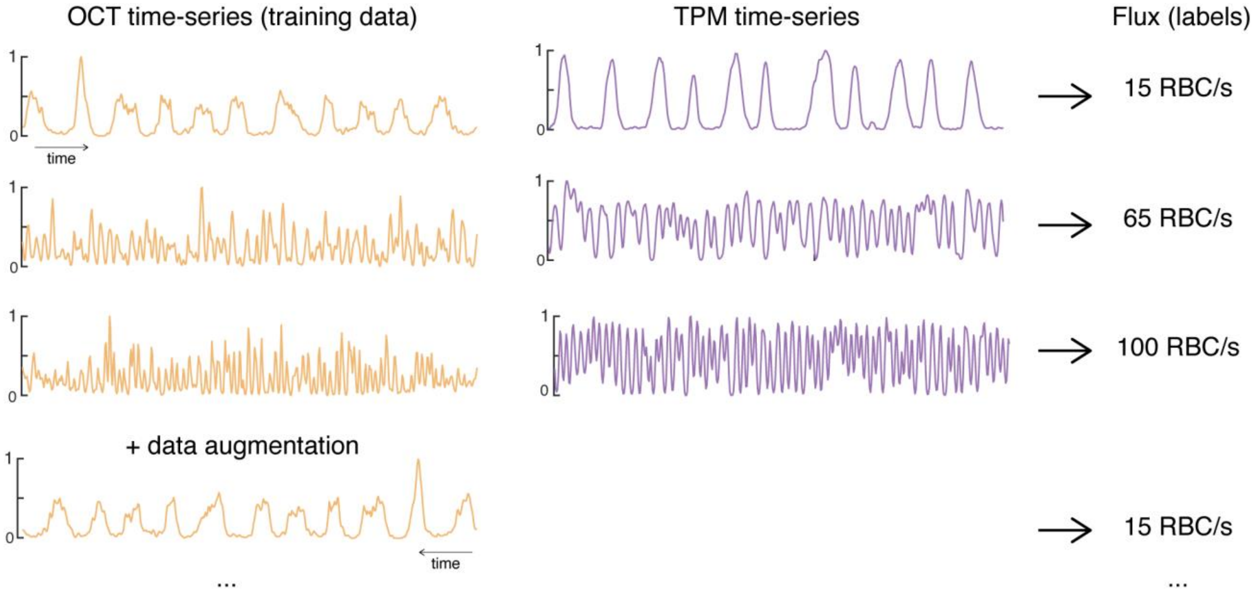

Supplementary Figure 5. Examples of time-series of RBC passages acquired simultaneously using OCT and TPM for low, average, and high flux values. The TPM time-series were used to obtain the “ground truth” for RBC flux. The OCT data was additionally augmented by flipping the time axis, thereby doubling the total training data.

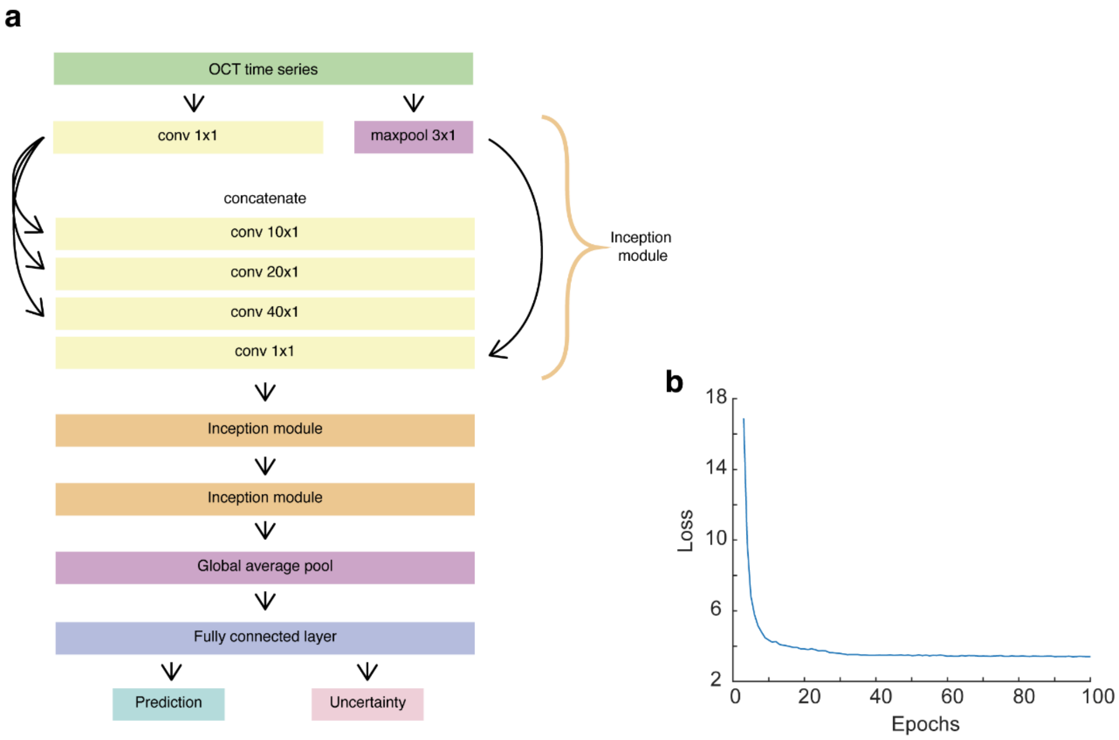

Supplementary Figure 6. **a)** InceptionTime 1D CNN architecture for the prediction of RBC flux from OCT time series data. **b)** The loss curve during the training of the CNN.

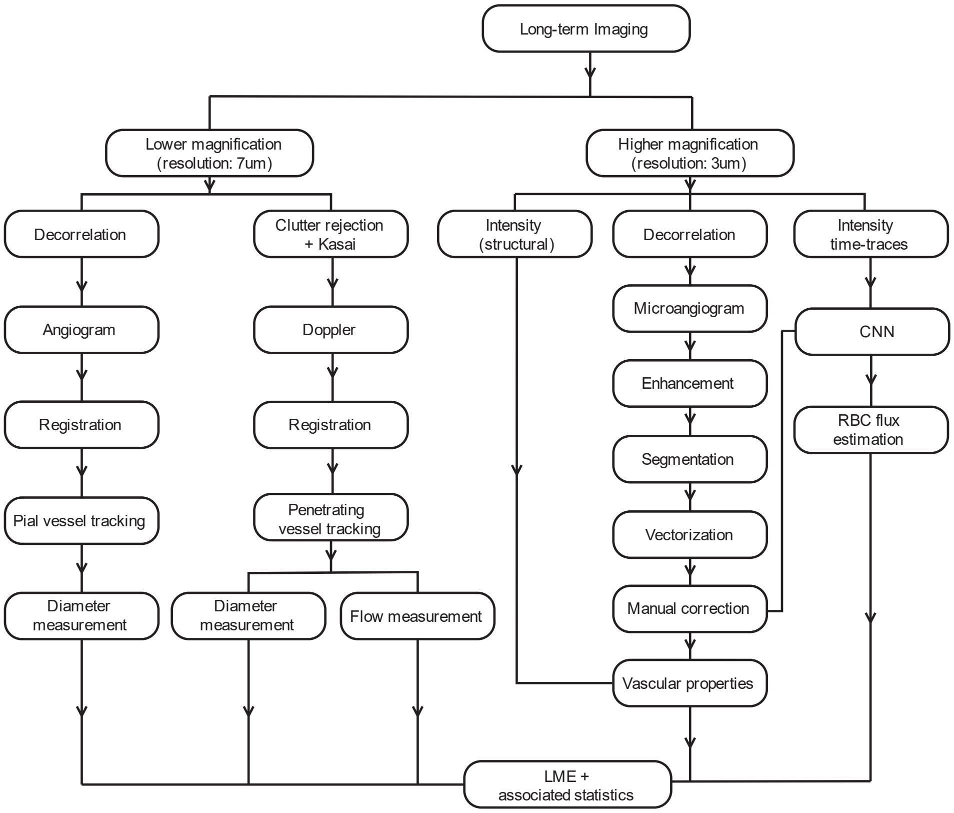

Supplementary Figure 7. Flowchart of analysis of longitudinal imaging data.

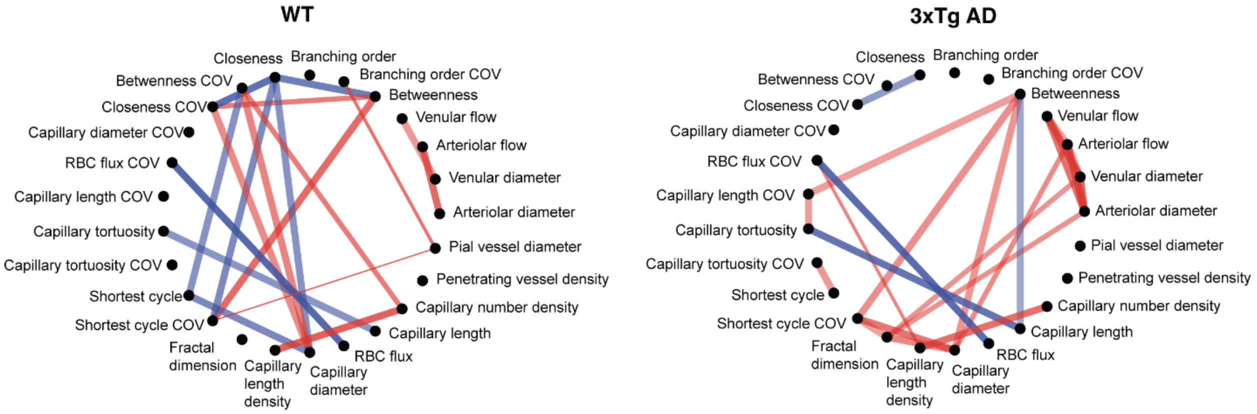

Supplementary Figure 8. Significant correlations among traces of vascular alterations ($p<0.05$ after Benjamini-Hochberg multi-hypotheses correction). The correlation was calculated between two time-courses of every possible pair, with age lags of 0, 4, 8, and 12 weeks. Pairs with positive and negative correlations are shown in red and blue respectively. More opaque lines indicate higher maximum correlation, and thinner lines means that the maximum correlation appeared at longer age lags. A correlation with an age lag means that one alteration was correlated to the other one with a certain time delay. Some correlations revealed here are unsurprising, such as the positive correlation between diameter and flow changes in the penetrating vessels, which is present in both AD and WT. Some other relationships may present new findings: for example, the betweenness formed the greatest number of connections with other vascular alterations, in both AD and WT, while it had the youngest age of significance among capillary vessel properties (Fig. 5c). This may shed light on a need for further investigation about the relatively novel topological property in aging research.

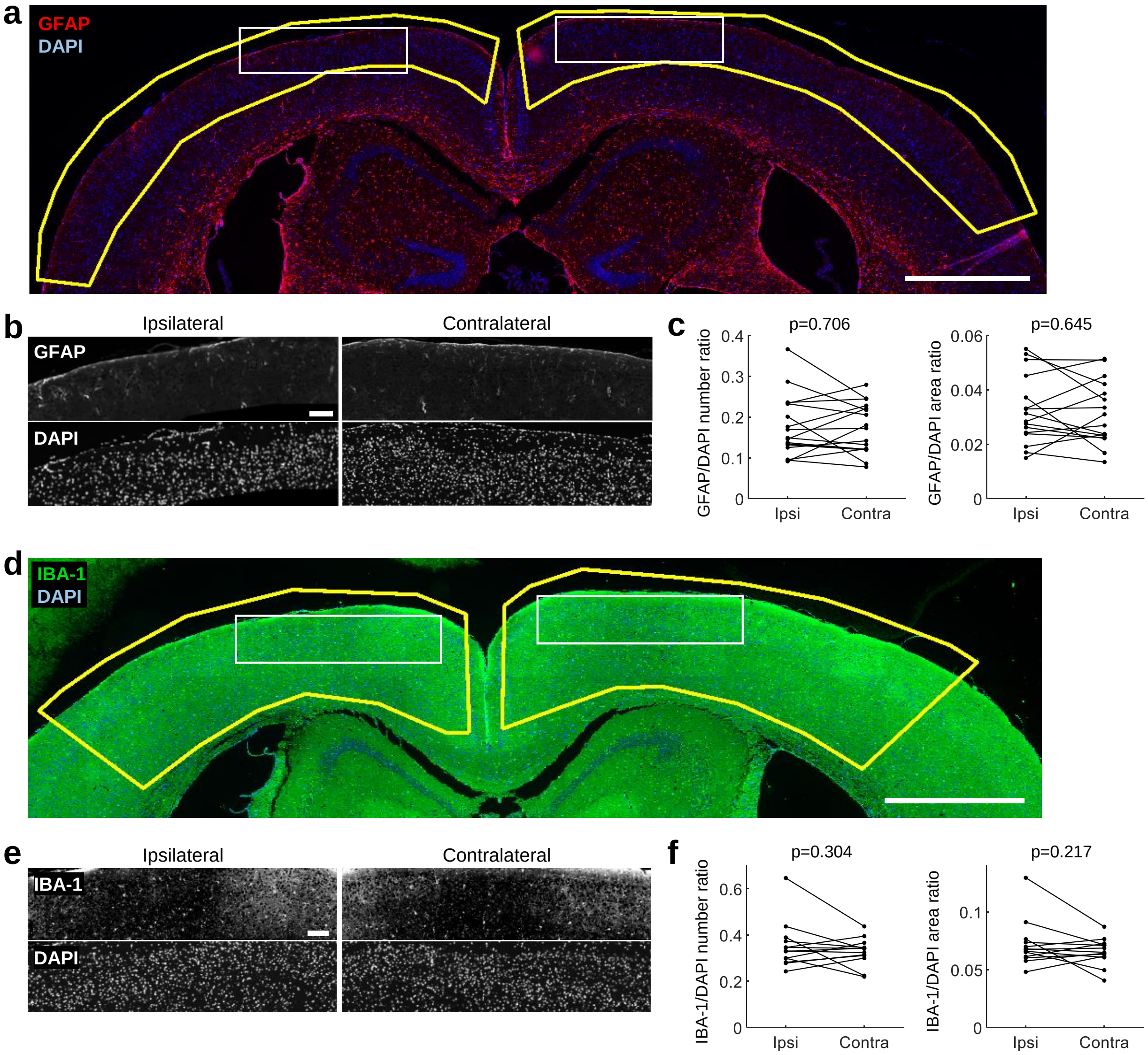

Supplementary Figure 9. Post-mortem immunofluorescence result. **a)** An example of GFAP and DAPI stain images. The yellow polygons indicate the regions of interest (ROIs) used for quantitative analysis in (c). Scale bar, 1 mm. **b)** Magnified images of the white rectangular areas in (a). Scale bar, 0.1 mm. **c)** 17 slices were analyzed (12 slices from 3 AD mice and 5 slices from 2 WT mice). All GFAP images underwent an identical image segmentation process, which used adaptive thresholding and filtered foreground objects by the area (equivalent to circles of 10-30 μm in diameter). DAPI images underwent circle detection processing. The differences were normally distributed, and we used paired, two-sided t-tests to analyze them. We excluded one slice as an outlier in the area ratio analysis. The effect of AD on this conclusion of insignificant

difference was not statistically significant (p=0.193, number ratio; 0.267, area ratio; linear mixed-effect [LME] analysis). **d)** An example of IBA-1 and DAPI stain images. The yellow polygons indicate the ROIs used for quantitative analysis in (f). Scale bar, 1 mm. **e)** Magnified images of the white rectangular areas in (d). The IBA-1 channel images underwent contrast adjustment to suppress background fluorescence. **f)** 13 slices were analyzed (9 slices from 2 AD and 4 slices from 1 WT mice). All IBA-1 images underwent the identical image segmentation as that for GFAP images. DAPI images also underwent the identical circle detection processing. The differences were normally distributed, and we used paired, two-sided t-tests to analyze them. The effect of AD on this conclusion of insignificant difference was not statistically significant (p=0.093, number ratio; 0.054, area ratio; LME analysis).

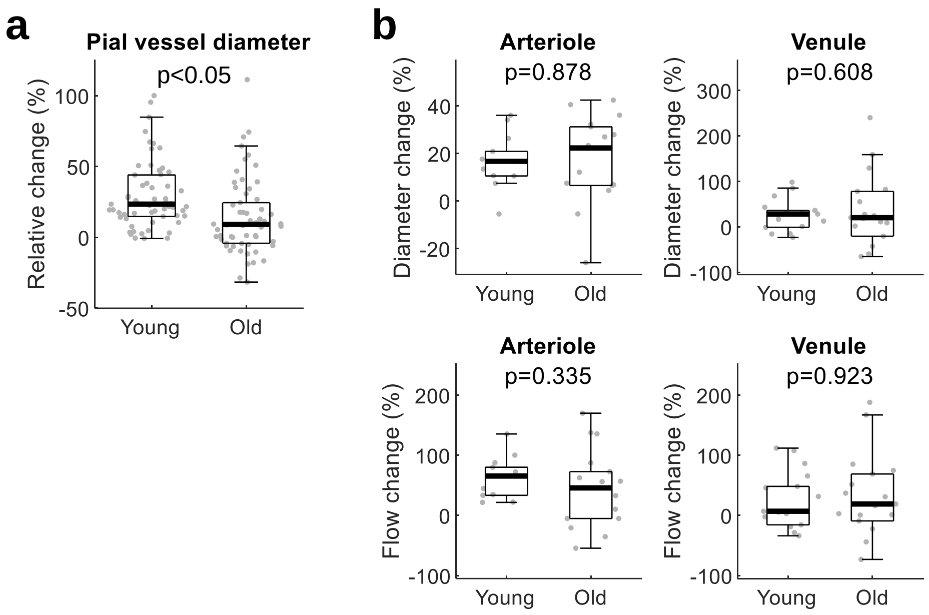

Supplementary Figure 10. Effects of isoflurane anesthesia on individual vessels. **a)** Effects of isoflurane on pial vessel diameter. 61 vessels (young) and 59 vessels (old) were measured and analyzed by bootstrapped LME, as traditional LME analysis produced non-normally distributed residuals. No multiple-comparison correction was needed. The bootstrapped LME, especially when bootstrapping the measures within each group, does not determine an exact p-value. **b)** Effects of isoflurane on arteriolar and venular diameter and flow. 11 arterioles and 15 venules (young) and 16 arterioles and 17 venules (old) were measured and analyzed by LME. Residuals were normally distributed. No multiple-comparison correction was needed. Each box chart displays the median (thick line), the lower and upper quartiles (box), and the minimum and maximum values that are not outliers (whiskers, computed using the 1.5x interquartile range).

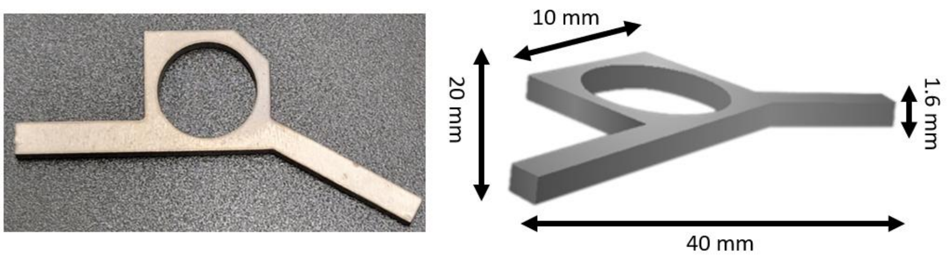

Supplementary Figure 11. Metal frame for head fixation during imaging. (Left) A picture of the metal frame used to affix the skull during the craniotomy surgery. The frame holds the head and minimizes motion artifacts during OCT imaging. (Right) The dimensions of the metal frame used in this study.

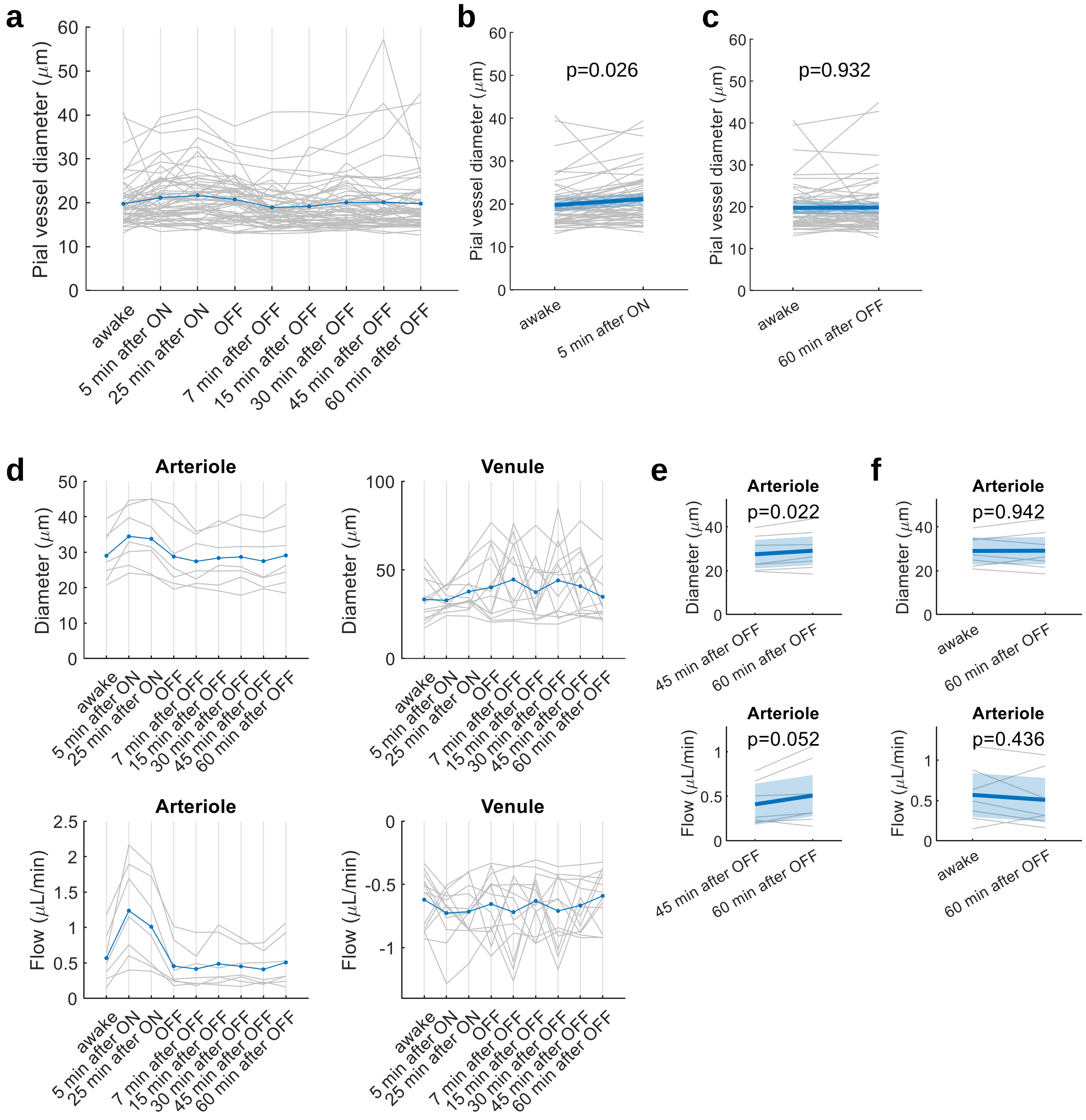

Supplementary Figure 12. OCT-measured changes in vascular diameter and flow during vasodilation and recovery. **a)** Tracked vessel diameter over 59 same pial vessels in four animals, with the blue line indicating the simple average of the diameters for each time point. **b)** Pial vessel diameter changes between the awake state and the state after 5 minutes of turning on isoflurane supply. The selected time points produced a p value close to 0.05. **c)** Pial vessel diameter changes between the first state (awake) and the final state (after 60 minutes of turning off isoflurane supply). **d)** Tracked vessel diameter and flow over seven same arterioles and 13 same venules in four animals. **e)** Arteriolar diameter and flow changes between the state before the final state (45 minutes after turning off isoflurane supply) and the final state, with the

selected time points producing p values close to 0.05. **f)** Arteriolar diameter and flow changes between the first and final states. In (b, c, e, f), the gray lines indicate individual vessels, the blue lines indicate LME fits, and the blue shades present the 95% CI. The p-values were obtained from the LME analysis (Wald test). No multiple-comparison correction was used.

Supplementary Table 1. Comparison of angioarchitecture properties available in the literature.

	This work (mean and 95% CI)	Ji et al.[1]	Kirst et al.[2]	Todorov et al.[3] (mean ± SE)	Blinder et al.[4] (median)	Schager et al.[5]
Branching order	3.7 (3 to 4)	3.4 ± 0.2 (mean ± SD)				
Capillary length (μm)	41 (33 to 48)				50	
Capillary tortuosity	1.27 (1.23 to 1.34)	1.27 ± 0.05 (mean ± SE)				~1.17
Shortest cycle	7.8 (5.3 to 10.3)	8.97 ± 0.53 (mean ± SE)			8	
Capillary length density (m/mm^{-3})	0.74 (0.67 to 0.80)	0.97 ± 0.03 (mean ± SE)	0.42	0.63 ± 0.09		~1.1

Supplementary Table 2. List of subfigures in previous work showing distributions of relevant properties.

	Gould et al.[6]	Haft-Javaherian et al.[7]	Ji et al.[1]	Blinder et al.[4]	Kirst et al.[2]
Branching order			Fig. 3g		Fig. 4i
Capillary length	Fig. 2b	Fig. 7	Fig. 5d	Fig. 2a	
Capillary tortuosity		Fig. 7	Fig. 3c		
Shortest cycle			Fig. 3e	Fig. 2d	
Capillary length density			Fig. 5b		

Supplementary Table 3. Effects of isoflurane anesthesia on vascular properties. Statistically significant values are presented in bold. The p-values were obtained using a Wald test. Bootstrapped LME does not provide an exact p-value. No multiple comparison correction was used.

Properties	Relative change (%, 95% CI) in young mice	Effect size of the older age (percent points, 95% CI) and its p-value	Sample size (young and old) and the used method
**Pial vessel diameter**	**24 to 35**	**-24 to -6 (p<0.05)**	61 and 59 vessels, bootstrapped LME
Arteriolar diameter	**7 to 27**	-12 to 14 (p=0.878)	11 and 16 vessels, LME
Venular diameter	**3 to 50**	-41 to 25 (p=0.608)	15 and 17 vessels, LME
Penetrating vessel density	-16 to 13	-19 to 22 (p = 0.893)	4 animals per group, bootstrap
Arteriolar flow	**24 to 108**	-86 to 31 (p=0.335)	11 and 16 vessels, LME
Venular flow	-5 to 60	-47 to 43 (p=0.923)	15 and 17 vessels, LME
Capillary length*	-8 to -2	-1 to 19 (p=0.341)	4 animals per group, bootstrap
Capillary length COV*	-9 to 4	-7 to 15 (p=0.522)	4 animals per group, bootstrap
Capillary diameter*	**1 to 23**	-18 to 14 (p=0.956)	4 animals per group, bootstrap
Capillary diameter COV*	**7 to 25**	-21 to 16 (p=0.483)	4 animals per group, bootstrap
Capillary tortuosity*	-3 to 5	-3 to 10 (p=0.422)	4 animals per group, bootstrap
Capillary tortuosity COV*	-34 to 55	-36 to 120 (p=0.336)	4 animals per group, bootstrap
Branching order*	-13 to 18	-20 to 23 (p = 0.854)	4 animals per group, bootstrap
Branching order COV*	-15 to 35	-49 to 21 (p = 0.413)	4 animals per group, bootstrap

Betweenness*	-24 to 30	-89.5 to -0.4 (p=0.184)	4 animals per group, bootstrap
Betweenness COV*	-21 to 21	-21 to 69 (p=0.426)	4 animals per group, bootstrap
Closeness*	-19 to 127	-104 to 209 (p=0.883)	4 animals per group, bootstrap
Closeness COV*	-3 to 20	-15 to 34 (p=0.785)	4 animals per group, bootstrap
Shortest cycle*	-6 to -1	-3 to 4 (p=0.839)	4 animals per group, bootstrap
Shortest cycle COV*	-3 to 11	-19 to 12 (p=0.807)	4 animals per group, bootstrap
Capillary number density	-2 to 2	-7 to 11 (p=0.384)	4 animals per group, bootstrap
Capillary length density	-1 to 2	-6.0 to 0.3 (p=0.305)	4 animals per group, bootstrap
Fractal dimension	-0.2 to 7.5	-8 to 3 (p=0.921)	4 animals per group, bootstrap
RBC flux*	**4 to 13**	-5 to 6 (p=0.563)	4 animals per group, bootstrap
RBC flux COV*	**-23 to -8**	-22 to 3 (p=0.326)	4 animals per group, bootstrap

* These capillary properties were measured and averaged (or calculated for COV) over 407-1718 capillaries within each animal (min-max) prior to being compared between the age groups.

Supplementary Text 1. Novelty statement

A variety of OCT imaging and image-processing techniques have been developed, adopted, and integrated for the presented framework. It ranges from reproduction of published methods (e.g., blood flow measurement from Doppler OCT data) and novel application of existing concepts (e.g., betweenness of a capillary network as adopted from graph theory) to development and validation of novel methods (e.g., the deep-learning method for capillary RBC flux measurement; Figs. 4a and 4b). But more importantly, the major novelty of the presented work lies in the integration of these existing and newly developed techniques into a single framework which has enabled to produce new types of information that otherwise would be highly challenging to obtain if not impossible (e.g., Figs. 5c and 5d).

Supplementary Text 2. Performance of the deep learning-based capillary RBC flux measurement.

Our CNN was also designed to provide a measure of uncertainty in each prediction, allowing us to filter out high-uncertainty predictions in further analysis. In contrast, the traditional peak-counting method does not provide the uncertainty of its estimation; thus, we used a single color on the left figure of Fig. 4a. This information about uncertainty was one of the many advantages of using the CNN-based method against the traditional peak-counting method. Even without this uncertainty filtering, the CNN outperformed the traditional peak-counting method when tested on data unseen by the CNN during training (Fig. 4a; the slope is closer to 1, and R^2 is higher). When filtering out predictions with the lowest 20% confidence, the error became both smaller (narrower distribution) and less biased (the mean closer to 0 RBC/s) even for higher flux values (Fig. 4b).

Regarding two-photon microscopy (TPM) data as the ground truth, TPM has been used as a standard method of measuring RBC flux and speed in capillary vessels of the rodent brain cortex[8–13]. It is based on fluorescence (staining plasma in most cases) and thereby yields a high signal-to-noise ratio. It is also capable of detecting RBC passage from vessels located deep in the cortical tissue, generally down to hundreds of micrometers deep, which cannot be achieved by

using an older approach based on video microscopy[14–16]. Thus far, to the best of our knowledge, no techniques offer more accurate measurements of RBC flux and speed than TPM.

Supplementary Text 3. Other challenges and opportunities related to the presented methods.

Differences in image quality and brightness may be unavoidable between imaging sessions across either different animals or ages. But many of the results obtainable by the presented methods, if not all, will offer true reflections of underlying physiology. Those image quality differences can originate from many technical and biological factors, including the angle of the beam with respect to the cranial window, the position of the focal plane, as well as the formation of scar tissue. Despite these factors, the presented results support statistical robustness against such random factors, or even against age-biased effects like a potential issue of subtle degrading image quality. Obvious degradation in image quality led to exclusion of those animals (3 out of 20, see Supplementary Text 8 for details). Potential subtle degradation of imaging quality which is not noticeable for exclusion, however, would lead to a loss of resolution and thus result in an apparent increase in the measured diameters of pial and penetrating vessels, but the observed results were opposite (Figs. 1e and 2d-2e).

A related challenge in a longitudinal imaging experiment with the cranial window is a limitation in employing behavioral tests to simultaneously perform on the same set of animals. In this study, we employed the NOL test alone for several reasons. First, the Morris water maze test, widely used in this context, could pose a risk of failure due to potential water damage to the cranial window headpost. Second, fear conditioning could be a potential addition to the NOL test, but repeatedly subjecting the same animal to conditioning and testing phases for several months could raise animal welfare concerns. Additionally, applying electric shocks to an animal with a metal head post attached could be a potential issue. Finally, the 3xTg model used in this study is one of the most widely used transgenic models of AD, and its cognitive decline has been well characterized in the literature. For example, Belfiore et al. found spatial learning and memory deficits at 6 months of age (26 WOA)[17], which is highly consistent with our result from the NOL test (27 WOA, Fig. 5b).

Another related challenge may be large variations in measurements between animals and/or ages. If one compared absolute values between groups age by age from the presented data, it might suffer from lower statistical power. Such age-by-age comparison does not exploit the analytic advantage of longitudinal data (Supplementary Fig. 1). Therefore, careful selection of a statistical method and clear focus of analysis are required. The LME method used here is often preferred for longitudinal data analysis over repeated ANOVA, as LME is relatively robust against missing values and violations of sphericity which increase rates of Type I error[18,19].

Another potential challenge in robustness is that the absolute values of diameters obtained from OCT angiogram (OCTA) may be inaccurate because the mechanism of OCTA contrast originates from the movement of RBCs through vessels. This may make the OCTA-measured diameter vary with measurement positions along the vessel direction. To minimize such variations, we measured the pial diameter from the mean of several cross-sections extracted along the vessel centerline. But this improves precision, not accuracy. Furthermore, isoflurane used in this study is known to influence vessel diameter. Despite these limitations in accuracy, high precision is often sufficient for many studies that focus on relative differences between experimental conditions rather than absolute values, like in the presented study. For a study where measurement of the absolute vessel diameter is critical, methods like fluorescent two-photon microscopy may be more suitable although its long-term longitudinal imaging capability has not yet been demonstrated to the best of our knowledge.

In addition to the properties measured in this study, a few related OCT measures have recently been proposed by others, and they can be readily added to the presented framework of methods. For example, capillary stalling has been found to play a role in pathology development in AD by a two-photon microscopy study[20] and then demonstrated to be measurable by OCT[21]. Also, it may be possible and interesting to study whether and how shear-induced diffusion of RBCs as measured by OCT[22] alters with aging.

Another potential opportunity of the presented method is to investigate subcortical vasculature when being used with a longer-wavelength OCT. We used the OCT with the center wavelength of 1.3 μm as it covers all cortical depths and the scope of this study focuses on cortical

microvasculature. OCT with longer wavelengths like 1.7 µm, however, can provide more homogeneous depth penetration and potentially reduce scattering, enabling further investigation of subcortical vasculature[23]. Combining the presented method with such a longer-wavelength OCT could enable researchers to explore how the vasculature beneath the cortex gradually alters in animal models of Alzheimer's disease, and how the alteration differs from that of cortical vasculature.

A direct methodological parallelism is another interesting potential of the presented methods. The OCT upon which the presented methods are based is already being widely used in clinical ophthalmology. Under the assumption that microvascular degenerations appear both in the brain and retina, some preclinical findings enabled by the presented methods may be directly applicable to human studies. In particular, a recent study suggests that cerebral microvascular degeneration may be one of the etiologies, independent of Aβ, in human ApoE4 AD[24]. ApoE4 accounts for a larger AD population than the familial AD for which the 3xTg model used in the present study was designed. Therefore, if the presented methods are applied to the ApoE4 AD model and produce a similar type of findings demonstrated here (e.g., the betweenness alters the earliest among capillary structural properties), and if such properties are measurable from human retinal OCT images, it would provide unprecedented potential for non-invasive, inexpensive, and imaging-based biomarkers for preemptive prediction of AD. Furthermore, such an application of the same method to both human and mouse will allow us to directly test any preclinical findings, a kind of ground truth comparison to animal models typically unavailable with most methods.

Supplementary Text 4. Use of the 3xTg AD model within the scope of the study.

First, it is important to note that the aim of this study is to demonstrate the technical capabilities of our approach as a preclinical research methodology, rather than directly addressing biological questions or developing a diagnosis technique for human AD. While Supplementary Text 3 briefly discussed the methodological parallelism and noted potential clinical applicability of our method, the validation of this applicability will require additional research, including bridge

studies such as cortical imaging of the ApoE4 model, retinal imaging of the ApoE4 model, and a small-scale proof-of-concept human study before conducting a large-scale clinical trial.

We chose the specific model because it quickly develops AD pathologies, since no studies have shown how long on the order of months the OCT methods can repeatedly and robustly image the cortex in the same animal through a chronic cranial window. Nevertheless, the results presented here may provide some insight to the roles of cerebral microvascular degeneration (CMD) in the development of AD, or at least showcases what type of findings will be possible when applied to appropriate models of broader age-related neurodegenerative diseases.

Since no rodent model fully captures the pathophysiology of human AD, a clear understanding of the strengths and weaknesses of each of the various AD models is required for proper interpretation[25]. Compared with some models like ApoE4 knock-in mice[26], the selected 3xTg model is not designed to directly develop CMD but focuses more on Aβ and tau pathologies[27,28]. However, CMD interacts with Aβ accumulation; Aβ often deposits on the vessel wall, leading to vascular dysfunction, while hypoperfusion accelerates Aβ accumulation[29]. Transgenic mice overexpressing the Aβ precursor protein have a profound and selective impairment in endothelium-dependent regulation of the neocortical microcirculation[30]. Therefore, vascular alterations observed from the 3xTg model may indicate either Aβ-associated vascular pathophysiology or independent CMD as a potential etiology of AD continuum[31,32].

While our study demonstrated that various vascular alterations precede cognitive decline and are correlated with the NOL test score, it does not establish causal evidence for the role of CMD in AD development. Rather, the results demonstrate the presented methods as a technological and data-driven approach for further research seeking to reveal the causal and mechanistic relationships between CMD and AD. For instance, by mitigating CMDs and monitoring their effects on long-term trajectories of neuropathology and cognitive impairment in diverse AD models, researchers could address whether CMD is an etiology or pathology of AD. The present study highlights some aspects of the cerebral microvasculature, or at least provides a means to generate related hypotheses, that could be further explored using techniques appropriate for testing causal relationships.

Lastly, it should be noted that the 3xTg model used in this study has little cerebral amyloid angiopathy, and thus, to specifically study vascular amyloid pathology, other models such as APP23 and triple transgenic Tg-SwDI models[33,34] may be employed. It would be interesting to compare the results presented here to those obtained from other models in future work.

Supplementary Text 5. Differences in the age of significance between arteriolar/venular diameter/flow.

The differences in the age of significance observed in Fig. 5c do not necessarily mean that arteriolar flow, for example, did not change while its diameter altered in early ages. As defined in the main text, the age of significance rather indicates the age at which a vascular alteration becomes pathological (different from WT, while the alteration can occur in both AD and WT), not indicating when the arteriolar flow becomes different from its baseline value (11 WOA). In fact, both arteriolar flow and diameter decreased in early ages (Figs. 2d and 2g). The difference in the age of significance only indicates that the alteration in the arteriolar diameter became pathological before the arteriolar flow alteration became pathological.

There are many models that explain the relationship between vascular structure and flow. In the most basic form commonly used in the literature, blood flow in a vessel can be modeled by Poiseuille's law[4,35]. This postulates that flow is, $F \propto \frac{r^4 \Delta P}{\eta L}$ where r is the vessel radius, ΔP is the pressure difference along the vessel, η is the viscosity and L is the vessel length. Flow is strongly related to the diameter of the vessel, with larger diameters increasing blood flow.

Based on this basic relation, one of the related compensation adaptations is likely the global autoregulation of cerebral blood flow (CBF). When the vessel radius decreases so that it tends to lower blood flow, the pressure can increase to maintain the blood flow at a similar level. This is a well-established principle of global autoregulation. Significant changes in CBF are not seen until this autoregulation is no longer able to compensate for the effect of structural degeneration, at which point flow begins to decrease[36].

Combining the morphology-flow model and the possible compensation adaptation, we can anticipate that since the compensating adaptation cannot be sustained indefinitely, eventually CBF would decrease due to the decreased vessel diameter. This explains one of our observations that blood flow decreased in penetrating vessels after the decrease in diameter.

Supplementary Text 6. Implication of the shortest cycle and betweenness result.

The result of these two independent topological measures (shortest cycle and betweenness, Figs. 3e and 3g) likely spot an identical type of CMD or at least different CMDs that later result in identical vascular vulnerability. The shortest cycle quantifies redundancy in a capillary network, while the betweenness measures the importance of each vessel to the connectivity of the whole network. A larger shortest cycle value means that a network is less redundant, while a higher mean betweenness implies that the network relies more on its subset of nodes (Supplementary Fig. 4). Disruption of flow in high-betweenness capillary vessels could have wide reaching effects (unless a physiological shunt of blood flow is established). In our results, both the shortest cycle and the mean betweenness increased with aging in AD, likely both indicating a higher degree of susceptibility to vascular insult than WT.

Supplementary Text 7. Interpretation of capillary length and flow results.

Our results showing a decrease in mean capillary length with aging in AD (Fig. 3d) are consistent with a previous study that found capillary length to be shorter in AD mice than in wild-type (WT) mice at 18-31 WOA[7], although that study did not longitudinally track the length and used a different AD mouse model. Another study found that capillary branch numbers were lower in 3xTg mice than WT mice at 20 months of age, but no difference was observed at 7 and 14 months[37]. This finding is consistent with our result of shorter capillary lengths in 3xTg mice, but our measurement revealed the difference much earlier (Fig. 3d). There are several possible reasons for this discrepancy, including that (i) the statistical analysis in the previous study did not consider the clustered nature of the data, leading to lower sensitivity to intra-group differences;

(ii) our method tracks the trend of the capillary property varying with age, whereas their method relies on a snapshot at a specific age; and/or (iii) our method analyzes 3D networks, while their method analyzes only 2D cross-sections of the network.

The pattern of blood flow through a capillary vessel network plays an important role in the energy supply regulation of the brain and its malfunction in diseases[38–41]. In our results, RBC flux gradually increased with aging in WT mice, being consistent with previous findings from WT rats[42] although the previous study measured RBC flux at only two ages in a non-longitudinal manner. AD mice also showed an increase in RBC flux but at a higher rate (6.0% versus 2.1% per month, Table 1). We also observed reduction in the COV of RBC flux in AD mice (i.e., more homogenized capillary flow pattern). Whereas higher RBC flux and lower flow heterogeneity in capillaries are generally associated with higher oxygen extraction[11,38], the observed increases in RBC flux and decreases in its heterogeneity with aging might be due to a compensatory mechanism for capillary vessels to suppress negative effects of the observed, earlier reduction in arteriolar and venular flow, similar to an adaptation occurring in hypoxia[43]. We speculate that such compensatory mechanisms do not last indefinitely; thus, if we had tracked the capillary flow properties for an extended period, we might have seen a decrease in capillary RBC flux and increase in RBC flux COV at a later age. Further studies are required to test this hypothesis.

The combined results of arteriolar diameter/flow and capillary flow suggest another possible consequence of decreased arteriolar diameter and flow, namely an increase in the presence of hypoxic micro-pockets in the cortex. The observed decreases in both arteriolar diameter and flow would lead to arteriolar and near-arteriolar tissue oxygen pressure (pO2)[44]. This impaired arteriolar oxygen delivery would not immediately lead to an impairment in capillary-bed tissue pO2 when the compensatory mechanism by enhanced capillary flow works as hypothesized above from our observed capillary RBC flux increases. However, when the compensatory mechanism no longer works at later ages, the capillary-bed tissue pO2 would become lower and spatially more heterogeneous, as observed between 60 and 100 WOA in WT mice[44]. This impaired capillary oxygen supply increases the presence of hypoxic micro-pockets, which may explain the observation of tinier microinfarcts in aging brains, particularly those with mild cognitive decline or AD[45,46]. Therefore, the decrease in arteriolar diameter and flow observed in

both WT and 3xTg mice with age may have important implications for the development and progression of neurodegenerative diseases.

The decrease in penetrating arteriole and venule diameters (Table 1) further suggests that the blood pressure might have increased with age in the 3xTg mice we used. Unfortunately, we did not longitudinally measure blood pressure in our study. Hypertension has been linked to AD, and animal studies suggest that hypertension can lead to amyloid plaques, neuroinflammation, blood-brain barrier dysfunction, and cognitive impairment[47]. However, human studies on the association between hypertension and AD have been sparse and inconsistent, and the effect of antihypertensive medications on AD seems weak[48,49]. Investigating interactions among plaques, tangles, cerebrovascular pathology, and dementia may be key to understanding hypertension's role in AD development[49]. Therefore, our future work includes performing a long-term trace of blood pressure using a noninvasive method like the tail-cuff technique in diverse AD models, which will enable us to compare the long-term blood pressure trace with the traces of cerebral microvascular properties obtained by the presented methods.

Supplementary Text 8. Potential impact of long-term craniotomy and imaging on animal physiology.

As described in the Methods section, we started with 20 animals, but one animal did not survive until the end of our seven-month longitudinal experiment. Three animals were euthanized in the middle of the study due to mechanical damage to their headposts, and three animals were excluded due to degrading image quality. The degrading image quality (3 out of 20) was first noticeable as blurry capillaries in microangiograms, likely due to the formation of a thin layer of scar tissue below the bottom surface of the glass and the top surface of the cortex. The mechanical damage (3 out of 20) occurred while the animals were moving in their cages. The spontaneous death rate (1 out of 20, 5%) was comparable to the known survival rate at the corresponding age[50]. This consistent survival rate suggests that chronic cranial windows might not have a severe long-term influence on the physiology of animals.

Several studies have investigated the effect of open-skull craniotomy on cortical physiology, particularly in terms of immunoreactivity. The literature suggests that the craniotomy may lead to higher immunoreactivity within 3-4 weeks after installation, but the immunoreactivity returns to normal after that time. For example, Xu et al. reported higher immunoreactivity in the window-installed cortex than the other side within 20 days of installation, but no difference at 30 days[51]. Holtmaat et al. also reported that astrocytes, not microglia, became more immunopositive in the window-installed cortex at 2 weeks but returned to normal at 4 weeks of window installation[52]. Similarly, Goldey et al. reported no difference between the ipsilateral and contralateral cortices of window installation at about 10 days[53]. Heo et al. found that a PDMS window installation led to a high microglia cell density, not astrocytes, at 1 week but the density returned to normal at 3 weeks[54].

Although the spontaneous death rate and existing literature suggest that long-term craniotomy is unlikely to have adverse effects on animal physiology, there has been no direct investigation into this possibility. To address this, we conducted post-mortem immunofluorescence imaging of the brains of animals that had undergone our longitudinal experiment. Using the published method of Heo et al.[54], we fixed, sectioned, and stained the brains with GFAP and DAPI (Supplementary Fig. 9a) or IBA-1 and DAPI (Supplementary Fig. 9d), which respectively visualize astrocyte and microglia immunoreactivity. These two stains are widely used in the literature to investigate the effect of craniotomy on cortical physiology[51–54]. We measured astrocyte immunoreactivity by calculating the ratio of GFAP to DAPI in terms of cell number or pixel area, and found no statistically significant differences between the ipsilateral and contralateral cortices of the window installation in both ratios (Supplementary Fig. 9c). We performed similar analysis on the DAPI and IBA-1 stained images and found no statistically significant differences in microglia immunoreactivity (Supplementary Fig. 9f).

Supplementary Text 9. Impact of anesthesia on OCT measurements of vascular morphology, topology, and flow.

The presented study conducted OCT imaging under isoflurane anesthesia, which is known to dilate cerebral vessels. While this vasodilation effect may vary between imaging sessions, it

would work as random noise and not affect the main conclusion of the study. This is because the study focused on comparing relative changes in the rate of change with age (RCA) between AD and WT groups, rather than absolute values. However, if the vasodilation effect is systematically different between younger and older mice, it could affect the accuracy of the RCA measurement. Even in this case, it would not change the main conclusion of the study, as the focus was on the statistical difference in RCA between the groups, rather than absolute RCA values.

Nonetheless, it would be valuable to identify which vascular properties are affected by isoflurane anesthesia in an age-related manner, and which are not. As no study has comprehensively investigated this for younger and older mice, an additional experiment was conducted in WT mice.

Methods: Seven days after installing a chronic cranial window on a mouse as described in the main text, we placed the mouse on an air-floating platform (Mobile Homecage, Neurotar) for a head-fixed, freely-walking awake imaging condition, while connecting the mouse nose to the isoflurane vaporizer. Under this awake condition, we acquired a similar set of OCT data as in the main study, including OCT angiogram, Doppler OCT, microangiogram, and RBC passage data. Then, we turned on the isoflurane supply (1.5% isoflurane with oxygen flow of 1 L/min), and after 30 minutes, we repeated the OCT data acquisition under this anesthetized condition. We repeated this experiment in young (15 WOA) and old (55 WOA) mice (n=4 mice per age group). This dataset underwent the same analysis as described in the main study to measure all the vascular properties listed in Table 1. For statistical analysis of properties tracked vessel by vessel (Supplementary Fig. 9), we used the LME method involving animal-specific random effects, excluding any outliers when a residual was more than three scaled median absolute deviations from the median. When this LME method resulted in non-normally distributed residuals, we used a bootstrapped LME method with a bootstrapping number of 2,000. Since this bootstrapped LME analysis, like other bootstrap methods, only provides confidence intervals (CIs), we considered it statistically significant ($p<0.05$) when the bootstrapped CI with alpha = 0.05 does not involve zero. In the statistical analysis of capillary properties, where we averaged values of a property over all capillaries within each animal, since we cannot track thousands of the same

capillaries, the sample size was small (4 animals per age group), so we used the bootstrap method to find the CI from 1,000 bootstraps.

Results: As summarized in Supplementary Table 3, isoflurane anesthesia at the concentration we used statistically significantly increased the pial vessel diameter, arteriolar diameter, venular diameter, arteriolar flow, capillary diameter, capillary diameter COV, capillary RBC flux, and capillary RBC flux COV in young mice. It was interesting to confirm that significant changes were observed only for those properties expected to be affected by the isoflurane-induced vasodilation and hyperperfusion, except for venular flow ($p=0.092$), whereas other structural properties did not exhibit statistically significant changes, as expected, including capillary length, tortuosity, betweenness, and closeness. This distinct result among capillary morphological, topological, and flow properties supports the rigor of our approach.

Returning to the focus of this additional experiment, whether the isoflurane effect is different between young and old mice, the effect size of the older age was statistically significant for pial vessel diameter only (Supplementary Table 3, Supplementary Fig. 9a). The isoflurane-induced increase in pial vessel diameter was smaller in old mice ($p<0.05$).

Discussion: The isoflurane-induced vasodilation effect on pial vessel diameter was greater in younger mice. Therefore, when tracking the pial vessel diameter with aging and measuring its slope (RCA) as we did in the main study, a negative RCA may be observed even if the animals do not exhibit physiological decreases in pial vessel diameter. As expected, our main study observed a small negative RCA in pial vessel diameter, with no statistically significant difference between AD and WT mice (-1.3%/month and -1.1%/month for AD and WT, respectively, $p=0.78$ between AD and WT, Table 1). This negative RCA is highly likely due to the age-dependent pial vessel dilation effect of isoflurane. The effect size of the older age was -15% on average (Supplementary Table 3) across approximately 10 months of age (between 15 WOA and 55 WOA). This effect size is expected to generate an artifact RCA of -1.5%/month, assuming that the age-dependent pial vessel dilation effect can be linearly interpolated between 15 and 55 WOA. However, this artifact RCA falls within the confidence intervals of the pial vessel

diameter RCAs of both AD and WT mice as measured in the main study (-2.4 to -0.2 and -2.2 to 0.0 %/month in AD and WT, respectively, Table 1).

Supplementary Text 10. Sensitivity of OCT measurements of changes in vascular diameter and blood flow.

Although the statistical results presented in the main text support that our approach was sensitive enough to reveal subtle differences in those vascular changes with age between AD and WT mice, we conducted a separate experiment to directly quantify the sensitivity of our approach of detecting changes in the average diameter and blood flow of pial vessels and penetrating arterioles.

Methods: We used isoflurane as a vasodilator and repeated OCT imaging while vessels went through dynamic changes via the dilation and recovery. In detail, seven days after we installed a chronic cranial window on a mouse as described in the main text, we put the mouse on an air-floating platform (Mobile Homecage, Neurotar) for a head-fixed, freely-walking awake imaging condition, while the mouse nose is connected to the isoflurane vaporizer. We acquired nine sets of OCT angiogram and Doppler OCT images, following the exact protocols described in the main text, at the following time points:

1. Awake as a baseline
2. 5 minutes after turning ON isoflurane supply
3. 25 minutes after turning ON isoflurane supply
4. Right after turning OFF isoflurane supply
5. 7 minutes after turning OFF isoflurane supply
6. 15 minutes after turning OFF isoflurane supply
7. 30 minutes after turning OFF isoflurane supply
8. 45 minutes after turning OFF isoflurane supply
9. 60 minutes after turning OFF isoflurane supply

This longitudinal dataset underwent the analysis process as described in the main text, in order to identify same vessels across time points and measure the pial vessel diameter, arteriolar diameter and flow, and venular diameter and flow, for each vessel and each time point. We repeated this

data acquisition and analysis in four young WT mice. For statistical analysis of a change between two states, we used linear mixed-effect (LME) analysis with the nested random effects of individual vessels in animals. This nested LME was identical to the LME analysis used in the main text.

Results: As expected, the pial vessel diameter increased with isoflurane and returned to the baseline after recovery (Supplementary Fig. 12a). Our approach was sensitive enough to detect a detectable 7% change in the pial vessel diameter between the first awake state and the state 5 minutes after turning on the isoflurane supply. The average pial vessel diameter in the awake state was 19.7 µm, and the increased diameter in the second state was 1.4 µm ($p=0.026$, $n=59$ vessels from four animals; Supplementary Fig. 12b). Our approach was also robust enough to produce statistically insignificant results between the first and the final states as physiologically expected ($p=0.932$, Supplementary Fig. 12c) while still detecting the 1.4-µm change.

Similarly, the arteriolar diameter and flow increased with isoflurane and returned to the baseline after recovery, while the venular diameter and flow did not (Supplementary Fig. 12d). We found subtle fluctuations during the recovery phase, the degree of which our approach was able to detect. The average arteriolar diameter in the first of the final two states (45 minutes after turning off isoflurane supply) was 27.5 µm, and the increased diameter in the final state was 1.6 µm, a 6% change ($p=0.022$, $n=7$ arterioles from four animals; Supplementary Fig. 12e, top). The average arteriolar flow changed by 0.098 µL/min from 0.409 µL/min, a 24% change ($p=0.052$, Supplementary Fig. 12e, bottom). Our approach was also robust in producing statistically insignificant results between the first and the final states as biologically expected ($p=0.942$ and 0.436 for diameter and flow, respectively; Supplementary Fig. 12f).

Discussion: The supplementary experiment presented here provides a quantitative measure of the sensitivity of our approach for detecting changes in vascular diameter and blood flow. We found the sensitivity of detecting changes in pial vessel diameter, arteriolar diameter, and arteriolar blood flow to be 1.4 µm (7% change), 1.6 µm (6% change), and 0.098 uL/min (24% change), respectively. It is important to note that these sensitivity values serve as a conservative estimate of the actual sensitivity of the measurements described in the main text. This is because the

supplementary experiment used fewer animals (four, against 6-7 per group of the main study) and compared only two time points, whereas the main experiment employed seven time points to determine the slope (rate of change with age, RCA). It is likely that the RCA measurement described in the main text has higher statistical power and thereby higher sensitivity.

Supplementary Text 11. Comparison to Aβ pathology in AD.

Various cerebral microvascular degenerations (CMDs) observed in this study became apparent between 12 and 25 WOA (Fig. 5c), preceding extracellular Aβ deposits in 3xTg model mice. In this specific model, extracellular Aβ deposits first become apparent in the frontal cortex at 6 months (26 WOA) and then evident in other cortical regions and in the hippocampus by 12 months (52 WOA)[27], and cortical Aβ plaques were first detected at 12 months of age (52 WOA)[17]. This temporal relationship between microvascular and Aβ pathologies in the 3xTg model agreed with findings from a recent study using the corrosion cast method[55]. It is difficult to directly compare results between this terminal study and our study, because the terminal study compared absolute values between AD and WT mice, age by age, while our longitudinal study compared the slope of relative changes (RCA) between AD and WT considering all ages of measurement. Nevertheless, we found some consistency. In the somatosensory cortex, the area we investigated, the terminal study found the total length of capillary vessels (5-10 μm in diameter) was higher at 3 months of age, similar at 6 months, and lower at 12 months in 3xTg compared to WT mice, which is consistent with our result of decreasing capillary length in 3xTg mice (Fig. 3d). The terminal study also found no difference in vessel segment and vessel junction numbers between 3xTg and WT mice across the ages of 3-24 months. This result is agreeable with our result of insignificant differences in capillary branching order, number density, and length density (Table 1). Finally, the terminal study observed more tortuous vessels in 3xTg mice, although it did not conduct quantitative analysis of tortuosity, and we also found an increasing capillary tortuosity with age in 3xTg mice (Table 1).

Supplementary references

1. Ji, X. *et al.* Brain microvasculature has a common topology with local differences in geometry that match metabolic load. *Neuron* **109**, 1168-1187.e13 (2021).
2. Kirst, C. *et al.* Mapping the Fine-Scale Organization and Plasticity of the Brain Vasculature. *Cell* **180**, 780-795.e25 (2020).
3. Todorov, M. I. *et al.* Machine learning analysis of whole mouse brain vasculature. *Nat. Methods* **17**, 442–449 (2020).
4. Blinder, P. *et al.* The cortical angiome: an interconnected vascular network with noncolumnar patterns of blood flow. *Nat. Neurosci.* **16**, 889–897 (2013).
5. Schager, B. & Brown, C. E. Susceptibility to capillary plugging can predict brain region specific vessel loss with aging. *J. Cereb. Blood Flow Metab. Off. J. Int. Soc. Cereb. Blood Flow Metab.* **40**, 2475–2490 (2020).
6. Gould, D. J., Vadakkan, T. J., Poché, R. A. & Dickinson, M. E. Multifractal and lacunarity analysis of microvascular morphology and remodeling. *Microcirc. N. Y. N 1994* **18**, 136–151 (2011).
7. Haft-Javaherian, M. *et al.* Deep convolutional neural networks for segmenting 3D in vivo multiphoton images of vasculature in Alzheimer disease mouse models. *PloS One* **14**, e0213539 (2019).
8. Kleinfeld, D., Mitra, P. P., Helmchen, F. & Denk, W. Fluctuations and stimulus-induced changes in blood flow observed in individual capillaries in layers 2 through 4 of rat neocortex. *Proc. Natl. Acad. Sci.* **95**, 15741–15746 (1998).

9. Schaffer, C. B. *et al.* Two-Photon Imaging of Cortical Surface Microvessels Reveals a Robust Redistribution in Blood Flow after Vascular Occlusion. *PLOS Biol.* **4**, e22 (2006).
10. Lecoq, J. *et al.* Simultaneous two-photon imaging of oxygen and blood flow in deep cerebral vessels. *Nat. Med.* **17**, 893–898 (2011).
11. Li, B. *et al.* More homogeneous capillary flow and oxygenation in deeper cortical layers correlate with increased oxygen extraction. *eLife* **8**, e42299 (2019).
12. Grubb, S. *et al.* Precapillary sphincters maintain perfusion in the cerebral cortex. *Nat. Commun.* **11**, 395 (2020).
13. Meng, G. *et al.* Ultrafast two-photon fluorescence imaging of cerebral blood circulation in the mouse brain in vivo. *Proc. Natl. Acad. Sci.* **119**, e2117346119 (2022).
14. Hudetz, A. G., Feher, G., Weigle, C. G., Knuese, D. E. & Kampine, J. P. Video microscopy of cerebrocortical capillary flow: response to hypotension and intracranial hypertension. *Am. J. Physiol.-Heart Circ. Physiol.* **268**, H2202–H2210 (1995).
15. Kim, S., Popel, A. S., Intaglietta, M. & Johnson, P. C. Effect of erythrocyte aggregation at normal human levels on functional capillary density in rat spinotrapezius muscle. *Am. J. Physiol.-Heart Circ. Physiol.* **290**, H941–H947 (2006).
16. Tomita, M., Tomita, Y., Unekawa, M., Toriumi, H. & Suzuki, N. Oscillating neuro-capillary coupling during cortical spreading depression as observed by tracking of FITC-labeled RBCs in single capillaries. *NeuroImage* **56**, 1001–1010 (2011).
17. Belfiore, R. *et al.* Temporal and regional progression of Alzheimer’s disease-like pathology in 3xTg-AD mice. *Aging Cell* **18**, e12873 (2019).

18. Gueorguieva, R. & Krystal, J. H. Move over ANOVA: progress in analyzing repeated-measures data and its reflection in papers published in the Archives of General Psychiatry. *Arch. Gen. Psychiatry* **61**, 310–317 (2004).

19. Muller, K. E. & Barton, C. N. Approximate Power for Repeated-Measures ANOVA Lacking Sphericity. *J. Am. Stat. Assoc.* **84**, 549–555 (1989).

20. Cruz Hernández, J. C. *et al.* Neutrophil adhesion in brain capillaries reduces cortical blood flow and impairs memory function in Alzheimer's disease mouse models. *Nat. Neurosci.* **22**, 413–420 (2019).

21. Erdener, Ş. E. *et al.* Spatio-temporal dynamics of cerebral capillary segments with stalling red blood cells. *J. Cereb. Blood Flow Metab. Off. J. Int. Soc. Cereb. Blood Flow Metab.* **39**, 886–900 (2019).

22. Tang, J. *et al.* Shear-induced diffusion of red blood cells measured with dynamic light scattering-optical coherence tomography. *J. Biophotonics* **11**, (2018).

23. Chong, S. P. *et al.* Noninvasive, in vivo imaging of subcortical mouse brain regions with 1.7 μm optical coherence tomography. *Opt. Lett.* **40**, 4911–4914 (2015).

24. Montagne, A. *et al.* APOE4 leads to blood-brain barrier dysfunction predicting cognitive decline. *Nature* **581**, 71–76 (2020).

25. Drummond, E. & Wisniewski, T. Alzheimer's disease: experimental models and reality. *Acta Neuropathol. (Berl.)* **133**, 155–175 (2017).

26. Knouff, C. *et al.* Apo E structure determines VLDL clearance and atherosclerosis risk in mice. *J. Clin. Invest.* **103**, 1579–1586 (1999).

27. Oddo, S. *et al.* Triple-transgenic model of Alzheimer's disease with plaques and tangles: intracellular Abeta and synaptic dysfunction. *Neuron* **39**, 409–421 (2003).

28. Billings, L. M., Oddo, S., Green, K. N., McGaugh, J. L. & LaFerla, F. M. Intraneuronal Abeta causes the onset of early Alzheimer's disease-related cognitive deficits in transgenic mice. *Neuron* **45**, 675–688 (2005).

29. Zlokovic, B. V. Neurovascular pathways to neurodegeneration in Alzheimer's disease and other disorders. *Nat. Rev. Neurosci.* **12**, 723–738 (2011).

30. Iadecola, C. *et al.* SOD1 rescues cerebral endothelial dysfunction in mice overexpressing amyloid precursor protein. *Nat. Neurosci.* **2**, 157–161 (1999).

31. Liu, P.-P., Xie, Y., Meng, X.-Y. & Kang, J.-S. History and progress of hypotheses and clinical trials for Alzheimer's disease. *Signal Transduct. Target. Ther.* **4**, 29 (2019).

32. Jack, C. R. *et al.* NIA-AA Research Framework: Toward a biological definition of Alzheimer's disease. *Alzheimers Dement. J. Alzheimers Assoc.* **14**, 535–562 (2018).

33. Castellani, R. J., Smith, M. A., Perry, G. & Friedland, R. P. Cerebral amyloid angiopathy: major contributor or decorative response to Alzheimer's disease pathogenesis. *Neurobiol. Aging* **25**, 599–602; discussion 603-604 (2004).

34. Miao, J. *et al.* Cerebral microvascular amyloid beta protein deposition induces vascular degeneration and neuroinflammation in transgenic mice expressing human vasculotropic mutant amyloid beta precursor protein. *Am. J. Pathol.* **167**, 505–515 (2005).

35. Schmid, F., Tsai, P. S., Kleinfeld, D., Jenny, P. & Weber, B. Depth-dependent flow and pressure characteristics in cortical microvascular networks. *PLOS Comput. Biol.* **13**, e1005392 (2017).

36. Claassen, J. A. & Zhang, R. Cerebral autoregulation in Alzheimer's disease. *J. Cereb. Blood Flow Metab.* **31**, 1572–1577 (2011).

37. Hohsfield, L. A., Daschil, N., Orädd, G., Strömberg, I. & Humpel, C. Vascular pathology of 20-month-old hypercholesterolemia mice in comparison to triple-transgenic and APPSwDI Alzheimer's disease mouse models. *Mol. Cell. Neurosci.* **63**, 83–95 (2014).

38. Jespersen, S. N. & Østergaard, L. The roles of cerebral blood flow, capillary transit time heterogeneity, and oxygen tension in brain oxygenation and metabolism. *J. Cereb. Blood Flow Metab. Off. J. Int. Soc. Cereb. Blood Flow Metab.* **32**, 264–277 (2012).

39. Gutiérrez-Jiménez, E. *et al.* Effect of electrical forepaw stimulation on capillary transit-time heterogeneity (CTH). *J. Cereb. Blood Flow Metab. Off. J. Int. Soc. Cereb. Blood Flow Metab.* **36**, 2072–2086 (2016).

40. Lee, J., Wu, W. & Boas, D. A. Early capillary flux homogenization in response to neural activation. *J. Cereb. Blood Flow Metab. Off. J. Int. Soc. Cereb. Blood Flow Metab.* **36**, 375–380 (2016).

41. Gutiérrez-Jiménez, E. *et al.* Disturbances in the control of capillary flow in an aged APPswe/PS1ΔE9 model of Alzheimer's disease. *Neurobiol. Aging* **62**, 82–94 (2018).

42. Desjardins, M., Berti, R., Lefebvre, J., Dubeau, S. & Lesage, F. Aging-related differences in cerebral capillary blood flow in anesthetized rats. *Neurobiol. Aging* **35**, 1947–1955 (2014).

43. Hudetz, A. G., Biswal, B. B., Fehér, G. & Kampine, J. P. Effects of hypoxia and hypercapnia on capillary flow velocity in the rat cerebral cortex. *Microvasc. Res.* **54**, 35–42 (1997).

44. Moeini, M. *et al.* Compromised microvascular oxygen delivery increases brain tissue vulnerability with age. *Sci. Rep.* **8**, 8219 (2018).

45. Smith, E. E., Schneider, J. A., Wardlaw, J. M. & Greenberg, S. M. Cerebral microinfarcts: the invisible lesions. *Lancet Neurol.* **11**, 272–282 (2012).

46. Wang, M. *et al.* Cognitive deficits and delayed neuronal loss in a mouse model of multiple microinfarcts. *J. Neurosci. Off. J. Soc. Neurosci.* **32**, 17948–17960 (2012).

47. Carnevale, D. *et al.* Hypertension induces brain β-amyloid accumulation, cognitive impairment, and memory deterioration through activation of receptor for advanced glycation end products in brain vasculature. *Hypertens. Dallas Tex 1979* **60**, 188–197 (2012).

48. Lennon, M. J., Koncz, R. & Sachdev, P. S. Hypertension and Alzheimer's disease: is the picture any clearer? *Curr. Opin. Psychiatry* **34**, 142–148 (2021).

49. Abdulrahman, H. *et al.* Hypertension and Alzheimer's disease pathology at autopsy: A systematic review. *Alzheimers Dement. J. Alzheimers Assoc.* **18**, 2308–2326 (2022).

50. Kane, A. E. *et al.* Sex Differences in Healthspan Predict Lifespan in the 3xTg-AD Mouse Model of Alzheimer's Disease. *Front. Aging Neurosci.* **10**, 172 (2018).

51. Xu, H.-T., Pan, F., Yang, G. & Gan, W.-B. Choice of cranial window type for in vivo imaging affects dendritic spine turnover in the cortex. *Nat. Neurosci.* **10**, 549–551 (2007).

52. Holtmaat, A. *et al.* Long-term, high-resolution imaging in the mouse neocortex through a chronic cranial window. *Nat. Protoc.* **4**, 1128 (2009).

53. Goldey, G. J. *et al.* Long-term imaging in awake mice using removable cranial windows. *Nat. Protoc.* **9**, 2515–2538 (2014).

54. Heo, C. *et al.* A soft, transparent, freely accessible cranial window for chronic imaging and electrophysiology. *Sci. Rep.* **6**, 27818 (2016).

55. Quintana, D. D. *et al.* Microvascular degeneration occurs before plaque onset and progresses with age in 3xTg AD mice. *Neurobiol. Aging* **105**, 115–128 (2021).
