# Supplementary material for: Near-lifespan longitudinal tracking of brain microvascular morphology, topology, and flow in male mice
Source: Nat Commun. 2023 May 24;14:2982. doi: 10.1038/s41467-023-38609-z (PMC10205707; doi:10.1038/s41467-023-38609-z)
Supplement: Supplementary file 3 — Reporting Summary [file 41467_2023_38609_MOESM3_ESM.pdf]

nature portfolio

Corresponding author(s): Jonghwan Lee

Last updated by author(s): Apr 20, 2023

Reporting Summary

Nature Portfolio wishes to improve the reproducibility of the work that we publish. This form provides structure for consistency and transparency in reporting. For further information on Nature Portfolio policies, see our Editorial Policies and the Editorial Policy Checklist.

Statistics

For all statistical analyses, confirm that the following items are present in the figure legend, table legend, main text, or Methods section.

n/a	Confirmed	
☐	☒	The exact sample size (n) for each experimental group/condition, given as a discrete number and unit of measurement
☐	☒	A statement on whether measurements were taken from distinct samples or whether the same sample was measured repeatedly
☐	☒	The statistical test(s) used AND whether they are one- or two-sided<br>*Only common tests should be described solely by name; describe more complex techniques in the Methods section.*
☐	☒	A description of all covariates tested
☐	☒	A description of any assumptions or corrections, such as tests of normality and adjustment for multiple comparisons
☐	☒	A full description of the statistical parameters including central tendency (e.g. means) or other basic estimates (e.g. regression coefficient) AND variation (e.g. standard deviation) or associated estimates of uncertainty (e.g. confidence intervals)
☐	☒	For null hypothesis testing, the test statistic (e.g. F, t, r) with confidence intervals, effect sizes, degrees of freedom and P value noted<br>*Give P values as exact values whenever suitable.*
☒	☐	For Bayesian analysis, information on the choice of priors and Markov chain Monte Carlo settings
☒	☐	For hierarchical and complex designs, identification of the appropriate level for tests and full reporting of outcomes
☒	☐	Estimates of effect sizes (e.g. Cohen's d, Pearson's r), indicating how they were calculated

Our web collection on statistics for biologists contains articles on many of the points above.

Software and code

Policy information about availability of computer code

Data collection	Custom LabVIEW codes were used for collecting data, on LabVIEW version 2017.
Data analysis	Custom MATLAB codes were used for analyzing data, on MATLAB version R2019a. https://github.com/optobrain/adp-1-3xtg-cortex

For manuscripts utilizing custom algorithms or software that are central to the research but not yet described in published literature, software must be made available to editors and reviewers. We strongly encourage code deposition in a community repository (e.g. GitHub). See the Nature Portfolio guidelines for submitting code & software for further information.

Data

Policy information about availability of data

All manuscripts must include a data availability statement. This statement should provide the following information, where applicable:

- Accession codes, unique identifiers, or web links for publicly available datasets
- A description of any restrictions on data availability
- For clinical datasets or third party data, please ensure that the statement adheres to our policy

The raw image data is larger than 50 terabytes in size, difficult to be shared online, but all the measured property values used in statistical analysis can be found at https://doi.org/10.6084/m9.figshare.19178885.

Human research participants

Policy information about studies involving human research participants and Sex and Gender in Research.

Reporting on sex and gender	N/A
Population characteristics	N/A
Recruitment	N/A
Ethics oversight	N/A

Note that full information on the approval of the study protocol must also be provided in the manuscript.

Field-specific reporting

Please select the one below that is the best fit for your research. If you are not sure, read the appropriate sections before making your selection.

- [x] Life sciences
- [ ] Behavioural & social sciences
- [ ] Ecological, evolutionary & environmental sciences

For a reference copy of the document with all sections, see nature.com/documents/nr-reporting-summary-flat.pdf

Life sciences study design

All studies must disclose on these points even when the disclosure is negative.

Sample size	The sample size (n=10 per group) was determined by a power analysis with α=0.05 and β=0.20 based on the assumption that the group difference is larger than 200% of the in-group standard deviation, while considering the long-term survival rate of mice with chronic craniotomy in the literature (up to 60-70%).
Data exclusions	During the described LME analysis, outliers were excluded based on Cook's distance; any value with a Cook's distance greater than 3 times the mean was considered an outlier. Prior to image processing, we excluded low-quality images when it is visually very clear, without considering their groups and ages (e.g., 11 out 91 images were excluded due to a very low quality in micrangiogram processing).
Replication	Since the presented data were obtained from the seven-month longitudinal experiment, it was very difficult to, so we did not, obtain a separate longitudinal dataset to replicate the findings.
Randomization	The animals were allocated into the disease and wild-type groups based on their genotypes. The phenotype was validated via the novel object location test of cognitive impairment. We started the longitudinal imaging at the same age across all animals in both groups, to control the major covariate (age).
Blinding	Our image processing, feature extraction from the processed images, and statistical analysis of the extracted features were performed automatically by software codes. These codes did not have any group-specific algorithms, and identical processing and analysis parameters were used between the two groups. As a result, the data analysis was conducted in an effectively blinded manner. Although the investigators were not blinded to group allocation during data collection, standardized protocols were followed, ensuring consistency across all animal groups. Furthermore, the data analysis was independently performed by different investigators, ensuring that any potential bias during data acquisition, if present, would not influence the interpretation of the results.

Reporting for specific materials, systems and methods

We require information from authors about some types of materials, experimental systems and methods used in many studies. Here, indicate whether each material, system or method listed is relevant to your study. If you are not sure if a list item applies to your research, read the appropriate section before selecting a response.

Materials & experimental systems

n/a	Involved in the study	
[x]	[ ]	Antibodies
[x]	[ ]	Eukaryotic cell lines
[x]	[ ]	Palaeontology and archaeology
[ ]	[x]	Animals and other organisms
[x]	[ ]	Clinical data
[x]	[ ]	Dual use research of concern

Methods

n/a	Involved in the study	
[x]	[ ]	ChIP-seq
[x]	[ ]	Flow cytometry
[x]	[ ]	MRI-based neuroimaging

Animals and other research organisms

Policy information about studies involving animals; ARRIVE guidelines recommended for reporting animal research, and Sex and Gender in Research

Laboratory animals	Male wild-type (WT, C57BL/6J, Jackson Lab) mice (n = 10) and male 3xTg AD mice (B6;129-Tg(APPSwe,tauP301L)1Lfa Psen1TM1Mpm/Mmjax, Jackson Lab, n = 10) were used from 10 weeks of age.
Wild animals	The study did not involve wild animals.
Reporting on sex	The study was performed exclusively on male mice, as its primary focus was the technical demonstration of novel methods rather than on biological findings. The main objective was to establish and validate these methods, which are applicable to both sexes. We opted to use male mice in this study to maintain homogeneity among the experimental subjects, thereby reducing potential variability and confounding factors associated with sex differences. This decision enabled us to focus on the effectiveness and accuracy of the novel methods being developed. Although we acknowledge that the findings presented in the paper may be directly applicable to male mice only, we would like to emphasize that the methods developed in this study can be extended and applied to both male and female mice in future research.
Field-collected samples	The study did not involve samples collected from the field.
Ethics oversight	All experimental procedures involving animals were reviewed and approved by the Institutional Animal Care and Use Committee (IACUC) of Brown University and Rhode Island Hospital.

Note that full information on the approval of the study protocol must also be provided in the manuscript.
